# The effects of asthma on the oxidative stress, inflammation, and endothelial dysfunction in children with pneumonia

**DOI:** 10.1186/s12887-022-03596-5

**Published:** 2022-09-08

**Authors:** Ali Arjmand Shabestari, Fatemeh Imanparast, Pegah Mohaghegh, Habibeh Kiyanrad

**Affiliations:** 1 Department of Pediatrics, Amirkabir Hospital, Arak University of Medical Sciences, Arak, Iran; 2 Department of Biochemistry and Genetics, Faculty of Medicine, Arak University of Medical Sciences, Arak, Iran; 3 Community and Preventive Medicine Specialist, Faculty of Medicine, Arak University of Medical Sciences, Arak, Iran

**Keywords:** Community-acquired pneumonia, Asthma, Oxidative stress, Inflammation, Endothelial dysfunction

## Abstract

**Background:**

In community-acquired pneumonia (CAP), pulmonary vascular endothelial dysfunction, inflammation, and oxidative stress (OS) are prominent and interesting as the unfavorable clinical outcomes of it. Asthma as a common chronic respiratory disease may affect the clinical outcomes of pneumonia, but the exact mechanism of this effect remains unclear. The present study aimed to assess the effects of asthma on the OS, inflammation, and endothelial dysfunction biomarkers in the children pneumonia.

**Methods:**

A cross-sectional study designed with a total of 75 children including both severe CAP and asthma (as group I), severe CAP alone (as group II), and healthy children (as group III) was conducted. Fasting blood samples were taken to the assay of serum malondialdehyde (MDA), total antioxidant capacity (TAC), tumor necrosis factor-alpha (TNF-α), soluble vascular cell adhesion molecule-1 (sVCAM-1)*,* and plasminogen activator inhibitor-1 (PAI*-*1)*.* The mean of anthropometric and biochemical parameters was compared by ANOVA and Tukey post-hoc test between groups.

**Results:**

We observed TAC levels in groups I and II (0.997 ± 0.22 and 1.23 ± 0.21 mmol/l, respectively) were significantly lower compared with group III (1.46 ± 0.19 mmol/l, *P* value < 0.001). It was significantly higher in group II than in group I (*P* value < 0.001). Also, we observed MDA and TNF-α levels in groups I (6.94 ± 1.61 μmol/l, 7.34 ± 2.23 pg/ml, respectively) and II (2.57 ± 0.40 μmol/l, 5.54 ± 1.84 pg/ml, respectively) were significantly higher compared with group III (1.89 ± 0.27 μmol/l, 3.42 ± 1.32 pg/ml, *P* value < 0.001, *P* value < 0.001, respectively).

VCAM-1 and PAI*-*1 levels as the endothelial dysfunction biomarkers were significantly higher in group I (1.5 ± 0.62 mmol/l, 10.52 ± 3.2 AU/ml, respectively) compared with groups II (1.06 ± 0.53 mmol/l and 8.23 ± 3.4 AU/ml; *P* value < 0.001, *P* value < 0.001, respectively) and III (0.6 ± 0.35 mmol/l and 2.39 ± 0.83 AU/ml; *P* value < 0.001, *P* value < 0.001, respectively). Also, VCAM-1 and PAI*-*1 levels were significantly higher in group II compared with groups III (*P* value < 0.001, *P* value < 0.001).

**Conclusions:**

Asthma can exacerbate the vascular dysfunction of pneumonia in children by increasing oxidative stress, inflammation, and endothelial dysfunction.

## Introduction

Pneumonia as an infection of the lungs caused by bacteria, viruses, fungi, and parasites that imposes significant costs for the health care system and exhibits the most common reason for the death of infectious origin [1]. In this disease, polymorphonuclear neutrophils and macrophages fight with microorganisms by using reactive oxygen species (ROSs) and lysosomal enzymes [2]. As a consequence of the pulmonary defense mechanism in inflammatory diseases such as pneumonia and asthma, oxidative stress (OS) at the systemic level may have a central role with adverse clinical outcomes of these diseases, such as the endothelial dysfunction (ED), exacerbation of inflammation, shortness of breath, and ultimately acute respiratory distress syndrome (ARDS) and death [3–5].

ED causes pulmonary edema due to the increased endothelial permeability. The activated endothelium mediates leukocyte binding to express the adhesion molecules for example vascular cell adhesion molecule-1 (VCAM-1) and intercellular adhesion molecule- 1 (ICAM-1). Upon leukocyte binding, these adhesion molecules activate endothelial cell signal transduction and then alter endothelial cell shape for the opening of passageways, through which leukocytes can migrate [6, 7].

The early childhood pneumonia has the long-term effects such as restrictive or obstructive lung diseases [8]. Studies have demonstrated that pneumonia can replicate and maintain infection in human macrophages, endothelial cells, and aortic smooth muscle cells and cause endothelial dysfunction and arthrosclerosis [9].

Asthma is the most common chronic respiratory disease in children, which is prevalent in developing countries. Although it cannot be considered a direct cause of pneumonia, children with asthma are more prone to develop more severe pneumonia due to previous lung damage. As a result, a child with asthma may have more severe symptoms and complications from pneumonia. Asthma may exacerbate the clinical outcomes of pneumonia, such as ED [10].

We are not aware of any studies on assessing the differences in OS, inflammation, and ED biomarkers in children with both asthma and pneumonia compared with children with pneumonia only. Therefore, the current study assessed the alterations in OS, TNF-α, and ED biomarkers in children with asthma and pneumonia, children with pneumonia only, and healthy children.

## Materials and methods

### Study design and participants

A cross*-*sectional study was conducted at the Pediatric Clinic of Amirkabir Hospital in Arak, Iran, from January 2019 to September 2019 with a total of 75 children with the age range of between 2 and 10 years. Due to the effect of seasons on the severity of asthma, a total of 75 children were sampled in 3 months of winter (January 2019 to March 2019). The minimum sample size was calculated using the formula $N=2\times \left({\left({Z}_{1-\left(\alpha /2\right)}+{Z}_{1-\beta}\right)}^2\times \left({SD}_1^2+{SD}_2^2\right)\right)/\left({\overline{X}}_1-{\overline{X}}_2\right)$ with 80% power, α = 0.05, SD_1_ = 0.1, SD_2_ = 0.07, d = 0.15 and based on the information of the study by Muravlyova et al. in that compared the mean of MDA in blood of patients in dependence of CAP severity. Calculated sample size was 25 in each group that 75 children were recruited in the study [11].

Pneumonia was defined as an acute pulmonary infiltrate evident on chest radiography with symptoms and signs of a lower respiratory tract infection, fever, cough, and purulent sputum. Pneumonia was confirmed with physical exams, microbiologic culture data, and Chest x-ray [12, 13]. According to the World Health Organization (WHO) pneumonia severity classification algorithm, sCAP in children approved with cough and/or difficult breathing, lower chest wall indrawing, fever > 37.5 °C, age-adjusted tachypnoea (respiratory rate ≥ 40) with chest wall recession and/or peripheral oxygen saturation [SpO2] < 92% in air. Microbiologic data were also used to diagnose sCAP.

The asthma of children based on GINA 2017 recommendations was confirmed by a physician via the symptoms of recurrent coughing, wheezing, and chest tightness. In children > 6 years of age, final approval was done by impulse oscillometry, spirometry or response to bronchodilator. Due to the difficulty in diagnosing asthma in children less than 6 years of age by lung function tests, the diagnosis of asthma in these children was based on the objective document of signs or convincing parent-reported symptoms of airflow obstruction including wheezing, difficulty breathing and cough that which were corrected with asthma therapy (short-acting ß2-agonists (SABA) such as albuterol).

The inclusion criteria were (1) established diagnosis of asthma and sCAP (2) signed informed consent to participate in the study given by the parents, (3) use only albuterol for treatment of asthma.

The exclusion criteria included (1) presence of congenital anomalies of the respiratory system approved by imaging, (2) cystic fibrosis approved by sweat testing, (3) acute (as pathogens, allergens, toxins, burns, and frostbite) and chronic inflammatory (in diseases such as heart disease, diabetes, cancer, arthritis, and bowel diseases) (4) constant contact with persistent heavy smoker (5) Chronic inflammatory systemic diseases like rheumatoid arthritis, systemic lupus erythematosus, and multiple sclerosis. Due to the possible adverse effects of corticosteroid therapy on biochemical factors of this study, asthmatic children taking steroid drugs were excluded from the study.

The control group (*n* = 25) was composed of healthy children through the diagnostic tastings listed above and were negative and anthropometric characteristics similar to the experimental group. With the consent of the parents, we used the extra blood samples of the children who went to the laboratory to assess their health and their health was confirmed by a pediatrician.

### Ethical and safety considerations

The present study was ethically approved by the Committee on Human Research, Publication and Ethics (CHRPE) at Arak University of Medical Sciences, Arak, Iran (IR.ARAKMU.REC.1397.3001). Informed consent to participate of children was obtained of parents.

### Biochemical assessments

Blood samples were taken from children admitted to the hospital during the course of acute sCAP with or without asthma. Sampling was done on the first day of hospitalization of the child with pneumonia in the hospital. Aliquot samples of serums were saved after centrifugation (20 min, 3000 rpm) at − 80 °C.

TAC and MDA were measured using the colorimetric assay kit (Kiazist, Iran).

PAI-1 was measured as an indicator of ED by ELISA kit (Germany, ZellBio, ZB-11159C-H9648). Also, VCAM-1, as another indicator of ED, was measured by the ELISA kit (France, Diaclone SAS, 25020).

Serum TNF-α was measured through the ELISA method according to the manufacture’s instruction (Biovendor, Germany, Cat≠ RAF128R)*.*

### Statistical analysis

The Kolmogorov–Smirnov test was employed to assay the normal distribution of continuous variables. Anthropometric and biochemical parameters were expressed by the mean and standard deviation. Categorical variables were described using frequency the one-way ANOVA test and Post Hoc tests were employed to compare anthropometric and biochemical factors between groups. All statistical analyses were performed using SPSS version 17 (SPSS, Chicago, IL, USA). The level of significance was set at *p* <  0.05.

## Result

Table 1 presents the children’s anthropometric variables. The medians of children’s age were 3.52 ± 1.38, 3.08 ± 1.32, and 3.36 ± 1.46 years, respectively with *P* value = 0.493 and ranging from 2 to 10 years. The medians of children’s weight were 14.74 ± 2.89, 14.19 ± 3.46, and 14.46 ± 3.59 kg, respectively with *P* value = 0.618. The medians of children’s height were 97.46 ± 12.19, 94.22 ± 11.12, and 96.08 ± 12.59 cm, respectively with *P* value = 0.635. The children’s BMIs for all 3 groups were − 2 < z-scores < 1.

**Table 1:** Anthropometric variables of the studied children

Variable	Children with pneumonia and asthma (group I) *n* = 25	Children with pneumonia (group II) *n* = 25	Healthy children (group III) *n* = 25	*P* value
Sex (M/F)	15/10	13/12	15/10	–
Age (years)	3.52 ± 1.38	3.08 ± 1.32	3.36 ± 1.46	0.493
Weight (kg)	14.74 ± 2.89	14.19 ± 3.46	14.46 ± 3.59	0.618
Height (cm)	97.46 ± 12.19	94.22 ± 11.12	96.08 ± 12.59	0.635
BMI (z-score)	−2 < z-scores < 1	− 2 < z-scores < 1	− 2 < z-scores < 1	

Data are presented as mean value and standard deviation (SD); Indicator the presence or absence of a significant difference between the values of similar variables in the three groups (*P* value), *M/F* Male/Female, *BMI* Body Mass Index

Results showed TAC levels in groups I and II (0.997 ± 0.22 and 1.23 ± 0.21 mmol/l, respectively) were significantly less than group III (1.46 ± 0.19 mmol/l), which was lower in group I than in group II. Also, MDA and TNF-α levels in groups I (2.57 ± 0.40 μmol/l, 6.94 ± 1.61 mmol/l, respectively) and II (2.11 ± 0.26 μmol/l, 5.54 ± 1.84 mmol/l, respectively) were significantly higher compared with group III (1.89 ± 0.27 μmol/l, 3.42 ± 1.32 mmol/l, respectively), which was significantly higher in group I than in group II (Table 2). VCAM-1 and PAI*-*1 levels as ED biomarkers were significantly higher in group I (1.5. ± 0.62 mmol/l and 10.52 ± 3.2 AU/ml, respectively) compared with groups II (1.06 ± 0.53 mmol/l and 8.23 ± 3.4 AU/ml, respectively) and III (0.6 ± 0.35 mmol/l and 2.39 ± 0.83 AU/ml, respectively). VCAM-1 and PAI*-*1 levels were significantly higher in group II compared with groups III (Table 2).

**Table 2:** Biochemical parameters in control, children with pneumonia, and children with pneumonia and asthma

Variable mean ± SD	children with pneumonia and asthma (group I) *n* = 25	children with pneumonia (group II) *n* = 25	Healthy children (group III) *n* = 25	*P* value For ANOVA
TAC (mmol/l)	0.99 ± 0.22	1.23 ± 0.21	1.46 ± 0.19	< 0.001
MDA (μmol/l)	2.57 ± 0.40	2.11 ± 0.26	1.89 ± 0.27	< 0.001
TNF-α (pg/ml)	6.94 ± 1.61	5.54 ± 1.84	3.42 ± 1.32	< 0.001
VCAM-1 (mmol/l)	1.5 ± 0.62	1.06 ± 0.53	0.6. ±0.35	< 0.001
PAI-I (AU/ml)	10.52 ± 3.2	8.23 ± 3.4	2.39 ± 0.83	< 0.001

Data are presented as mean value and standard deviation (SD); Indicator the presence or absence of a significant difference between the values of similar variables in the three groups (*P* value), *MDA* Malondialdehyde, *TAC* Total antioxidant capacity, *VCAM-1* Vascular cell adhesion molecule 1, *PAI-1* Plasminogen activator inhibitor-1, *TNF-α* Tumor necrosis factor alpha

All the variables that showed significant variation between the groups studied were presented the POST HOC analysis table separately as Table 3. *P* values for post hoc analysis show a pair wise comparison of the groups.

**Table 3:** Comparison of the Biochemical parameters in control, children with pneumonia, and children with pneumonia and asthma with Post hoc test

Variable	Category	*P* value for Tukey			ANOVA
		children with pneumonia and asthma (group I) *n* = 25	children with pneumonia (group II) *n* = 25	Healthy children (group III) *n* = 25	
TAC (mmol/l)	children with pneumonia and asthma (group I) *n* = 25		< 0.001	< 0.001	F:30.42
	children with pneumonia (group II) *n* = 25	< 0.001		0.001	
	Healthy children (group III) *n* = 25	< 0.001	0.001		
MDA (μmol/l)	children with pneumonia and asthma (group I) *n* = 25		< 0.001	< 0.001	F: 29.22
	children with pneumonia (group II) *n* = 25	< 0.001		0.052	
	Healthy children (group III) *n* = 25	< 0.001	0.052		
TNF-α (pg/ml)	children with pneumonia and asthma (group I) *n* = 25		0.008	< 0.001	F: 30.39
	children with pneumonia (group II) *n* = 25	0.008		< 0.001	
	Healthy children (group III) *n* = 25	< 0.001	< 0.001		
VCAM-1 (mmol/l)	children with pneumonia and asthma (group I) *n* = 25		0.027	< 0.001	F: 16.96
	children with pneumonia (group II) *n* = 25	0.027		0.006	
	Healthy children (group III) *n* = 25	< 0.001	0.006		
PAI-I (AU/ml)	children with pneumonia and asthma (group I) *n* = 25		0.011	< 0.001	F: 58.60
	children with pneumonia (group II) *n* = 25	0.011		< 0.001	
	Healthy children (group III) *n* = 25	< 0.001	< 0.001		

Data are presented as mean value and standard deviation (SD); Indicator the presence or absence of a significant difference between the values of similar variables in the three groups (*P* value); *MDA* Malondialdehyde, *TAC* Total antioxidant capacity, *VCAM-1* Vascular cell adhesion molecule 1, *PAI*-*1* Plasminogen activator inhibitor-1, *TNF-α* Tumor necrosis factor alpha

## Discussion

Present study showed that in children with sCAP, biomarkers of OS, inflammation, and ED were significantly higher than the healthy children, and it is also higher in the asthmatic children with pneumonia than in the non-asthmatic children. This is probably because asthma may exacerbate OS and inflammation in children with pneumonia.

Studies have shown the interaction between pneumonia and cardiovascular diseases (CVDs). According to the cohort study of Violi et al. 2019, patients with CVDs had a higher risk of CAP, and conversely, CVDs risk was intensified with CAP. In recent years, CVDs were considered as an outcome of patients admitted to hospital with pneumonia infection [14]. After recovery of CAP in addition to the period of the acute infection, there is still the risk of acute cardiovascular events due to systematic inflammation [15].

The initial stage of the molecular and cellular stages leading to CVDs is ED [16, 17]. OS and inflammation are the two main causes of its creation [18, 19]. Therefore, the inflammatory diseases as asthma and CAP with increased OS can lead to ED, and this ED may make a person susceptible to CVDs in the future. Studies indicate the underlying respiratory diseases such as asthma may be effective in the severity of pneumonia injuries. Asthma, whose main feature is chronic inflammation in the airway wall, increases the risk of pneumonia. Incidence of pneumonia was observed more in children with asthma than health children. Local and systemic cytokine responses in particular, pro inflammatory cytokines that activate innate immune neutrophils as IL-6 and IL-18 increase in CAP. The concentration of some of them persist high even after the acute phase of pneumonia. Also, Pro-inflammatory cytokines such as TNF-α, IL-1β, and IL-6 increase in asthma. These increasing inflammation and airway reactivity support the potential interaction between CAP and subsequent asthma attacks [20–23]. Therefore, due to increased inflammation and oxidative stress in asthma and pneumonia, there is a possibility of increased ED factors in asthma and pneumonia.

In the present study, TNF- *α* was significantly higher in children with pneumonia and asthma than pneumonia and healthy children. Studies indicate the inflammatory process associated with ED exacerbates the severity of the consequences of CAP [24]. Also, recent evidence suggests a critical role for pneumonia infection in the pathogenesis of atherosclerosis by exacerbating OS, inflammation, and ED. Increasing the pro-inflammatory cytokine TNF- *α* as a consequence of pneumonia and asthma induce ED by various mechanisms, such as increasing the endothelial permeability and reducing the endothelium- dependent relaxation. It increased vascular endothelial growth factor (VEGF) as the endothelial permeability mediator and diminishing the half- life of mRNA encoding for endothelial nitric oxide synthase and decreasing nitric oxide production [25, 26].

In our study, VCAM-1 and PAI-I as two biomarkers of ED were significantly higher in children with both pneumonia and asthma than the children with pneumonia only. Also, they were significantly more in children with pneumonia than healthy children. OS and inflammation are closely linked with each other. Inflammatory mediators lead to OS, and reciprocally, OS increases the production of inflammatory mediators with the activation of NF-kB and AP-1. NF-κB and AP-1 are involved in the activation of pro-inflammatory molecules, such as vascular cell adhesion molecule − 1 (VCAM-1) and PAI-I [27].

In 2015, Lin et al. indicated that TNF-α-induced VCAM-1 expression in human cardiac fibroblasts was mediated by the activation of NF-κB by c-Src-mediated transactivation of the EGF receptor (EGFR)/PI3K/Akt cascade [28]. ROSs regulate several cells signaling pathways, such as expression of VCAM-1, resulting in the release of inflammatory mediators [29].

In our study, an increase in OS, MDA and TNF-α levels and a decrease TAC level in children with pneumonia with and without asthma than healthy children were observed. These findings were supported by Zhang et al. (2018), Pikuza et al. (2012), Majewska et al. (2004) [30–32]. Muravlyova et al. (2016) showed that sCAP patients have more levels of oxidative proteins and MDA in erythrocytes than moderate CAP and healthy volunteers [11].

ROSs concentration and time of exposure are two determining factors in the effects of OS in the airway as well as in other organs. Due to damage in biomolecules and inducing intracellular signaling pathways by ROSs, more concentration and longer exposure of ROSs can lead to cell death by apoptosis [33]. Accordingly, studies show that the attenuation of OS alleviates the organ damage.

Zhang et al. in 2018 demonstrated the treatment of CAP patients with N-acetylcysteine (NAC) reduces MDA and increases TAC compared with those in the non-NAC group [30]. In asthma as a chronic inflammatory airway disease, OS exacerbates airway inflammation by inducing various pro-inflammatory moderators, boosting bronchial hyper responsiveness, exciting bronchospasm, and increasing mucin secretion [34].

One of the limitations of this study was the non-participation of the children with asthma alone.

## Conclusions

The OS, inflammation, and ED biomarkers in children with asthma and pneumonia were significantly higher than them in children with pneumonia without asthma, and healthy children. Asthma can exacerbate the vascular dysfunction of pneumonia in the children by increasing the oxidative stress, inflammation, and endothelial dysfunction.

## Data Availability

All data generated or analyzed during this study are included in this published article.
